# Comparison of flow pressures in different 3-way infusion devices: an in-vitro study

**DOI:** 10.1186/s13037-018-0165-1

**Published:** 2018-06-29

**Authors:** Jonathan Chua, Arun Ratnavadivel

**Affiliations:** Department of Anaesthesia, Nepean Hospital, Kingswood, NSW 2747 Australia

**Keywords:** Transducers, Pressure, Infusion pumps

## Abstract

**Background:**

The use of multiple infusions through one cannula is an increasingly common practice in anaesthesia. High pressures in the line often lead to occlusion alarms and pump disconnection. In this study, we aim to determine the pressures generated in common 3-way infusion devices, using simple low-cost equipment available and currently in use in the operating theatre environment.

**Methods:**

We compared three different common and commercially available 3-way infusion devices that allowed multiple infusions through one cannula in vitro. One with anti-siphon valves, one without valves, and serial extension sets with side ports connected in series. An invasive blood pressure transducer was used to monitor line pressure. Seven different infusion rates were used to simulate different infusions.

**Results:**

3-way infusion devices with anti-siphon valves have 5.5 times the infusion pressures compared to devices without valves (*P* < 0.001). The highest pressures obtained across all devices were at the highest flow rate studied (400 ml/hr); this was 243 mmHg in the 3-way device with anti-siphon valves, compared to only 44 mmHg in the 3-way device without valves and 36 mmHg in the serial extension sets. Serial extension sets have the lowest pressures across all flow rates when compared to 3-way devices without valves. (*P* = 0.0001).

**Conclusions:**

The presence of anti-siphon valves generate very high pressures in infusion lines that can contribute to occlusion alarm disconnection of a pump. However, when measured alone and in-vitro, these pressures are not sufficient to trigger occlusion alarms. There are 3-way infusion devices without anti-siphon valves that have lower line pressures, but clinicians should be aware of negative pressure scenarios which can lead to siphoning when using them.

## Background

The administration of multiple infusions via one intravenous cannula is becoming increasingly common in the practice of anaesthesia. High pressures in the line often lead to occlusion alarm and pump disconnection, which makes it important to understand what contributes to raised line pressures.

The advent of new drugs and introduction of target-controlled infusion (TCI) pumps has made total intravenous anaesthesia (TIVA) a growing technique over the last 20 years. It offers advantages over traditional inhalational anaesthesia, including: improved quality of emergence; reduced postoperative nausea and vomiting; and earlier discharge in outpatient surgery [1, 2, 3]. In these cases, multiple infusions consisting of intravenous (IV) fluids, propofol, and remifentanil are often administered through a single cannula.

In order to deliver a drug, infusion pumps generate a force to overcome resistive forces and venous pressure. When such pressures exceed a set limit, occlusion alarms sound and a protective disconnect of the pump motor is initiated to prevent harm to the patient. Hence, thoughtful setup of infusion systems including the lines to avoid high pressures is important to avoid pump failure. The impetus for this study arose from anecdotal reports of increased incidence of occlusion alarms from members of our department after the introduction of a new 3-way infusion device with anti-siphon valves. We aimed to study the pressures generated in common types of 3-way infusion devices, using simple low-cost equipment available and currently in use in the operating theatre environment.

## Methods

**Fig. 1:**
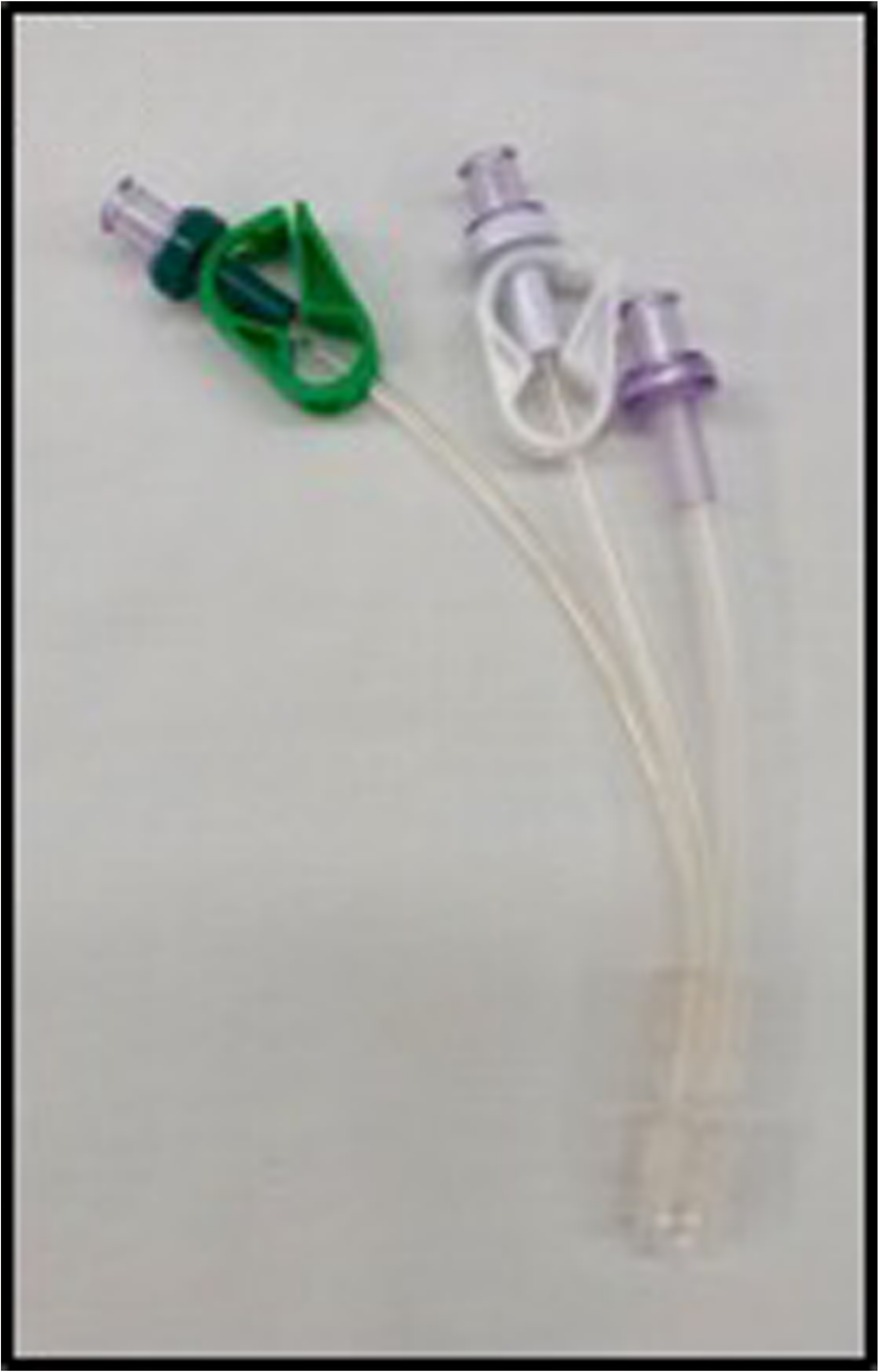
3-way device with anti-siphon valves

**Fig. 2:**
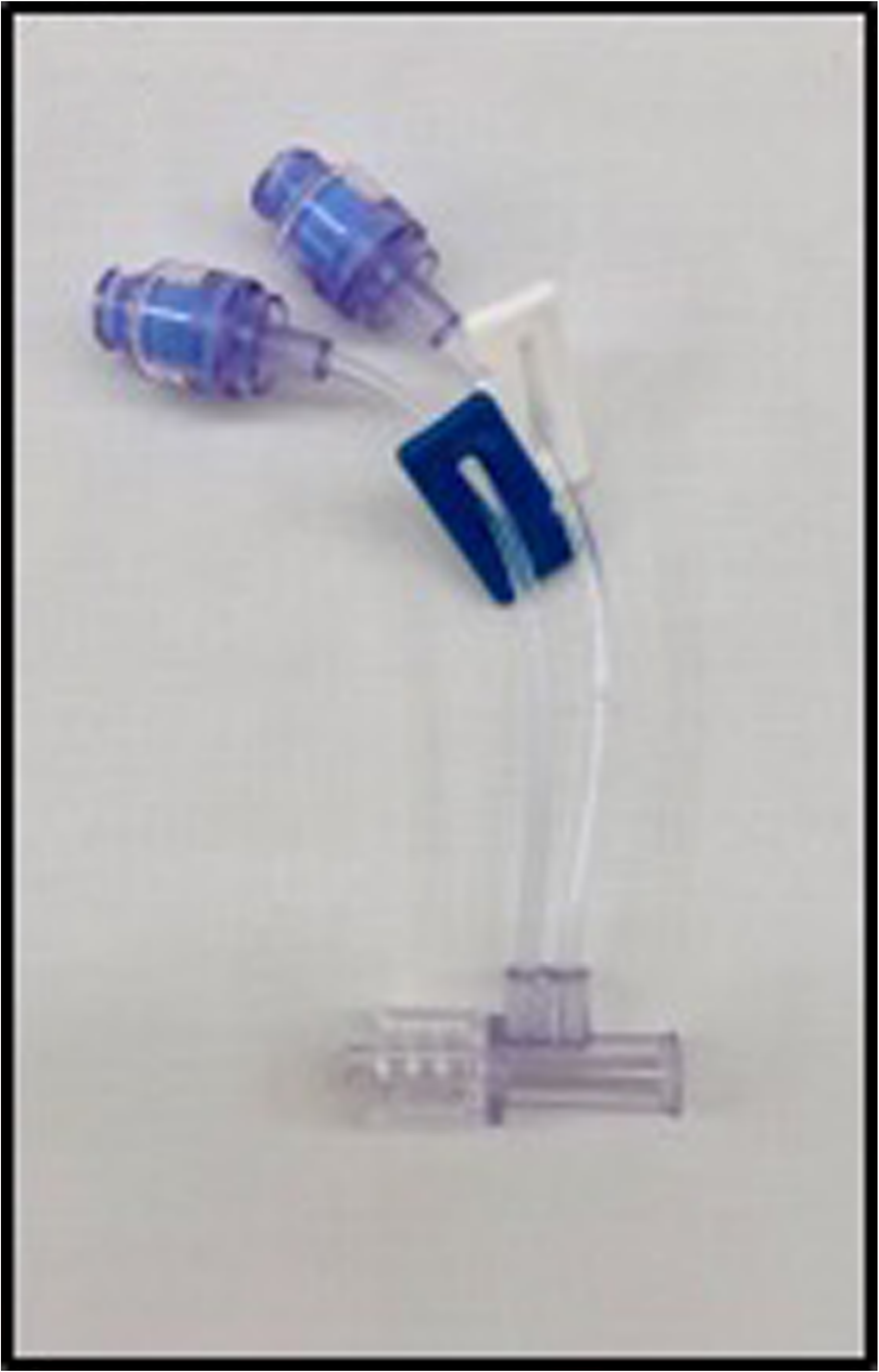
3-way device without valves

**Fig. 3:**
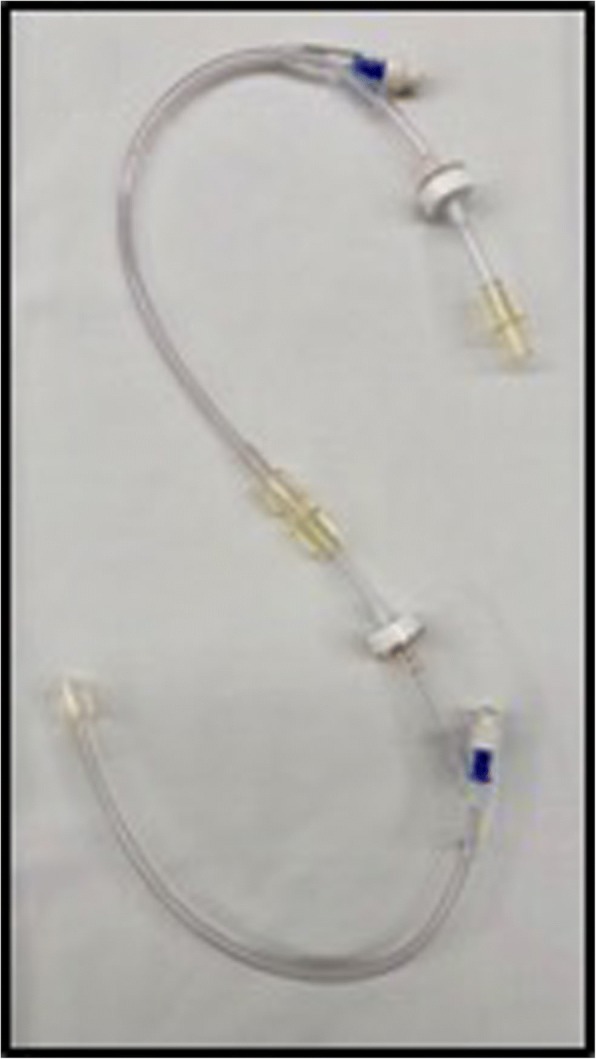
Two extension sets with side ports connected in series

**Fig. 4:**
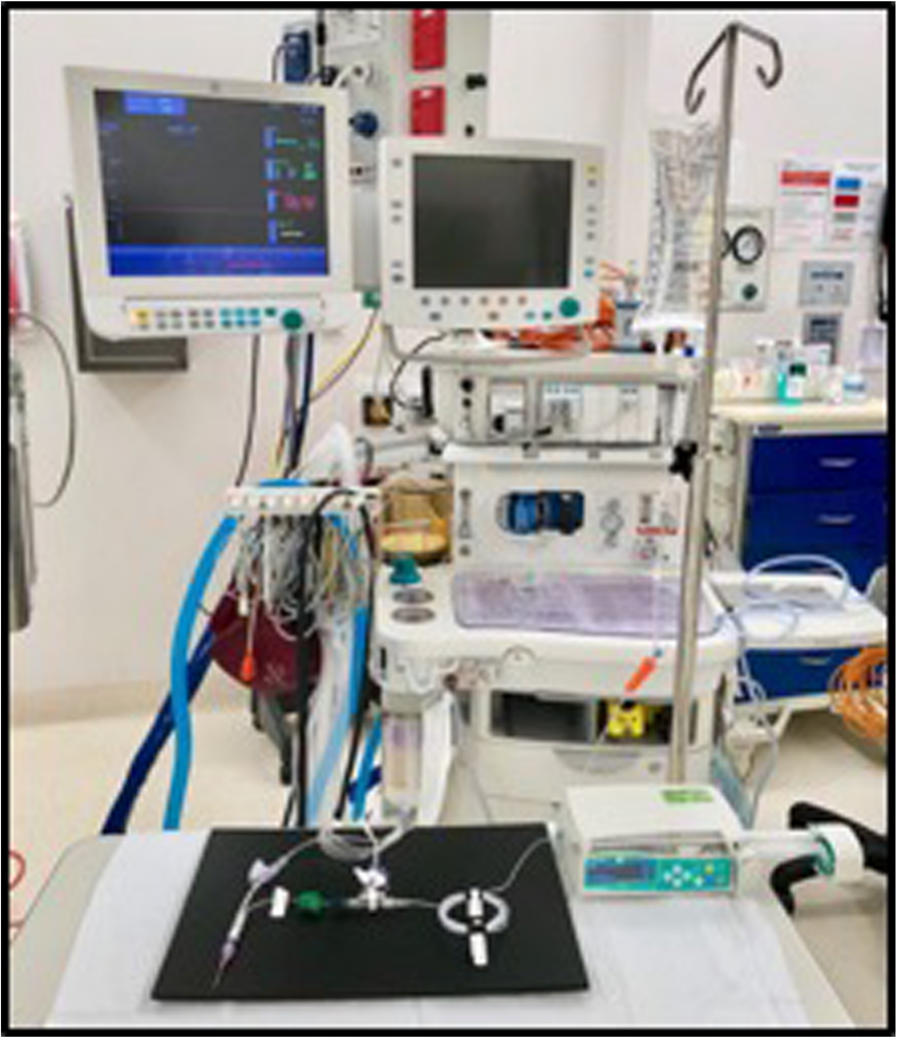
Full arrangement showing experimental setup connected to a syringe pump, bag of fluids, and the anaesthetic machine to display pressures

**Fig. 5:**
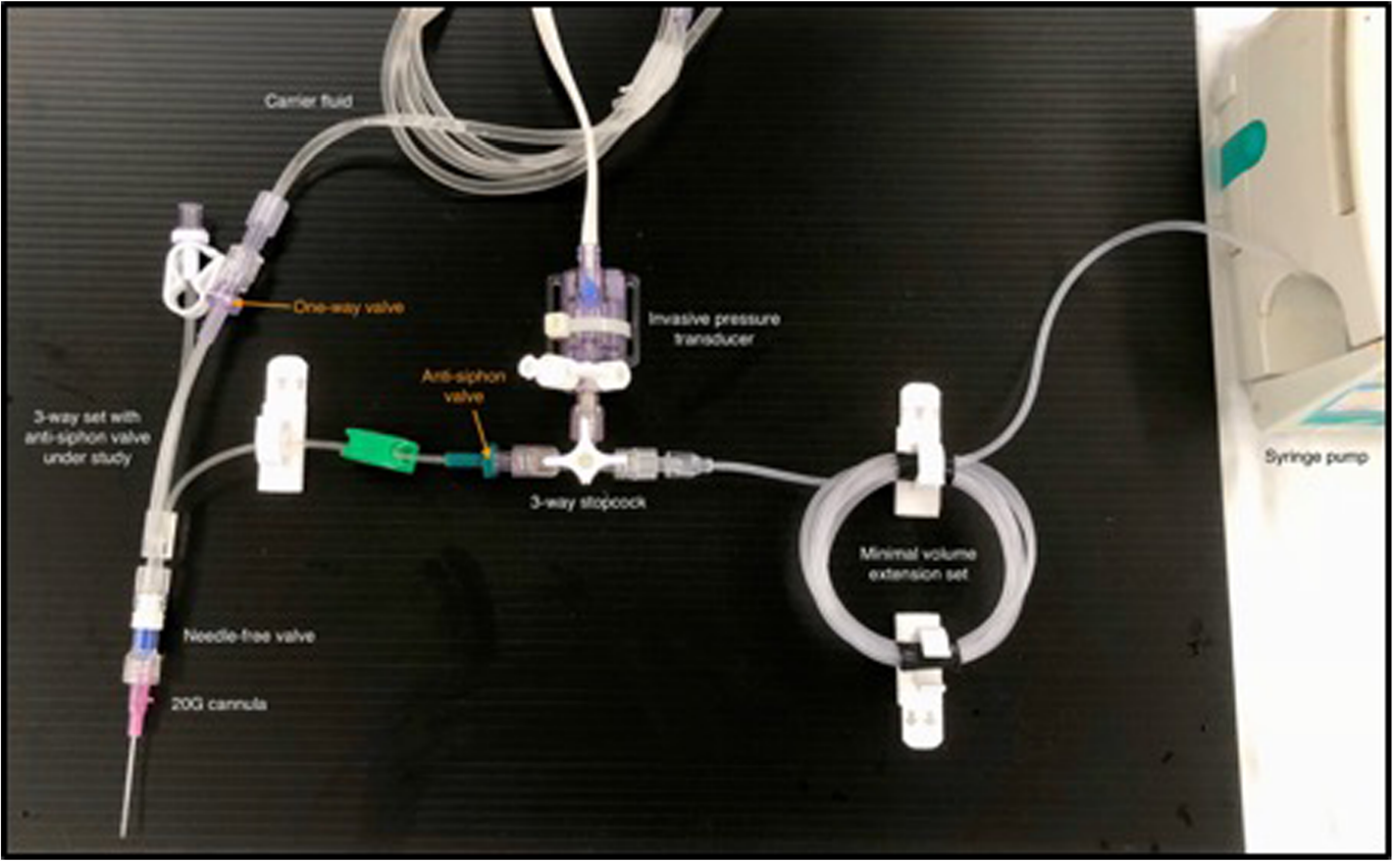
Close-up of experimental setup

We compared three different common and commercially available infusion devices that allowed multiple infusions through one cannula in vitro. A commercially available syringe infusion pump (Perfusor Space, B.Braun, Melsungen, Germany) containing a Luer lock 20 mL syringe (Terumo, Somerset, NJ, USA) was connected to a 205 cm extension set (Becton Dickinson, Franklin Lakes, NJ, USA). The extension line was then connected to a 3-way stopcock (Becton Dickinson). An invasive blood pressure transducer (TruWave, Edwards, Irvine, CA, USA) was connected to the middle stopcock to monitor line pressure. The distal stopcock was connected to the three different 3-way infusion devices. One with anti-siphon valves (500-003 V, Carefusion, Hampshire, UK); (Fig. 1), one 3-way device without valves (BC 6004, REM SYSTEMS, New Zealand); (Fig. 2), and serial extension sets with side ports connected in series (30842E, Becton Dickinson); (Fig. 3). A summary of their features is shown in Table 1. Each set was then connected to a needle-free valve (2000E, Carefusion) and 20G cannula (Introcan, B.Braun) opened to air. All components were positioned at the same level to eliminate any pressure effects of gravity. The experimental setup was chosen to reflect clinical practice and is shown in Figs. 4 and 5.

**Table 1:** Characteristics of the different infusion devices tested

Infusion device	Type of access	Anti-siphon valve?	One-way valve on fluid line?	Dead-space
A	3-way with anti-siphon valves	Yes	Yes	0.3–0.6 ml
B	3-way without valves	No	No	0.3 ml
C	Serial extension sets in series	No	Yes	2.4 ml

**Table 2:** Pressures encountered at different flow rates across the three devices tested

	Mean pressure (mmHg)		
Flow rate (ml/hr)	3-way with anti-siphon valves A	3-way without valves B	Serial extension sets C
10	178.7 ± 6	36.7 ± 1	32.7 ± 1
20	183.7 ± 7	37.0 ± 1	32.7 ± 1
30	185.7 ± 6	37.3 ± 1	32.7 ± 1
40	187.3 ± 5	37.3 ± 1	32.7 ± 1
50	189.7 ± 4	37.3 ± 1	32.7 ± 1
100	199.0 ± 1	38.7 ± 1	33.0 ± 1
400	243.3 ± 33	44.3 ± 1	35.7 ± 1

Seven different infusion rates were chosen to simulate the infusion of vasopressors, propofol, or remifentanil (10, 20, 30, 40, 50, 100, and 400 ml/hr). The syringe and lines were filled with normal saline, with care taken to evacuate all air bubbles. A simultaneous gravity infusion of carrier fluid was set up with normal saline running from a bag suspended 1 m above the setup. The pressure (“mean arterial pressure”) measured by the arterial line transducer was recorded at each flow rate after 60s of flow. Pressures at each flow rate was measured three times and the experiment repeated again with another set of 3-way devices. Results were analyzed using the paired t-test. All data are given as mean ± SD, with *P* values < 0.05 considered statistically significant.

## Results

**Fig. 6:**
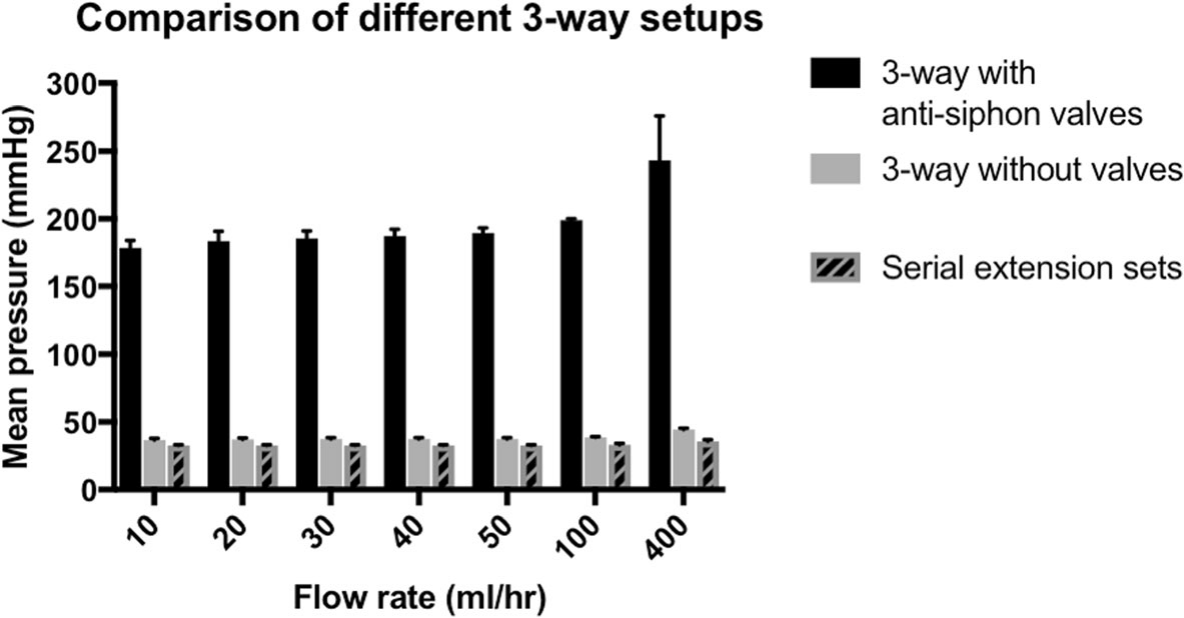
Pressures of different 3-way setups at different flow rates

3-way devices with anti-siphon valves (A) have much higher infusion pressures than devices without valves (B) (*P* < 0.001); (Fig. 6). The highest pressures obtained across all devices were at the highest flow rate; this was 243 mmHg in the 3-way device with anti-siphon valves (A), compared to only 44 mmHg in the 3-way device without valves (B) and 36 mmHg in the serial extension sets (C). Serial extension sets (C) have the lowest pressures across all flow rates when compared to 3-way devices without valves (B) (*P* = 0.0001).; (Fig. 6). Results are shown in Table 2. All results were repeatable with low standard deviations.

## Discussion

This is the first study to determine and compare the pressures in commonly used 3-way infusion devices using simple low-cost equipment employed in the operating theatre environment.

Our study reveals that the presence of anti-siphon valves increases infusion pressures considerably and proportionally to flow rate. These pressures are much higher than the quoted opening pressures of 100 – 150mmHg [4]. Across all flow rates, 3-way devices with anti-siphon valves developed approximately 5.5 times more pressure than devices without valves. Anti-siphon valves work by opening at a certain overpressure. Their importance is to prevent free flow of an infusion should negative pressure develop in the line. This can occur by positioning a disconnected syringe above the patient. Where there is a possibility of air entering the syringe, through cracks or an open stopcock, the level of the syringe needs only to be a few centimeters above the venous pressure to allow free flow of an infusion. This deleterious situation has been reported in the literature [4] with serious consequences to the patient. Despite the high pressures, we did not encounter occlusion alarms in-vitro with our pump occlusion alarm set at 500 mmHg, a default pressure used by many manufacturers. This suggests that a combination with other factors in-vivo is required for tripping the pump occlusion alarm. These are likely patient related, such as cannula positioning, and are beyond the scope of this study. Certainly, the presence of anti-siphon valves will reduce the threshold for the presence of these other factors. Technical data on the Perfusor Space pump reveal that occlusion pressures can be set between 75 and 900 mmHg. Indeed, if configured at the lowest setting, the pump would not work with the anti-siphon devices but will function with the other devices tested here.

Despite the lower pressures, the other devices studied have short-comings. The 3-way device without valves (B) do not have one-way valves along the gravity fluid port. Should the cannula be occluded, the contents of pump infusion will flow into the higher compliance fluid line. The pressure rise may take some time before occlusion alarms sound. Having one-way valves would reduce this delay and has been shown to increase the accuracy of drug delivery [5].

The serial extension sets (C) studied possess one-way valves and have the advantage of the lowest pressures. This can be attributed to a larger internal diameter. However, this brings about a total dead-space volume of 2.4 ml, compared to 0.3–0.6 ml in the other 3-way devices. Delay in reaching the targeted drug delivery rate has been shown to be directly related to dead-space volume [6]. Up to an extra 5 min lag time for drug delivery has been found in devices with high dead-space volumes [7].

Our study has some limitations. We did not use commercial pressure transducers. This has implications on the accuracy of low pressure measurement and limiting measurements to 320 mmHg. Because of this, we did not test higher flow rates (> 400 ml/hr) which generated pressures beyond what we could measure. The use of different infusion line arrangements, pumps, syringes, and cannula size would be expected to yield different pressures than those found in our study which limits its generalizability. Also, we used normal saline instead of specific drugs which may influence the frictional forces in the syringe and infusion lines. However, it remains likely that the highest pressure encountered in any infusion line is due to the presence of an anti-siphon valve.

## Conclusion

The ideal multi-infusion device would have the following properties: low resistance, low dead-space, anti-siphon valves, and one-way valves on carrier fluid lines. As we have seen from our study, the devices available and commonly used in practice have some but not all of these qualities. The implications for practice is the clinician must understand the limitations of the device being used. For example, if high occlusion pressures are encountered despite a functioning cannula and a line free from occlusion, a solution may be to change to a setup without anti-siphon valves. However, one must keep in mind that in doing so removes a layer of safety and must be vigilant against situations that can lead to siphoning as covered above.

In conclusion, anti-siphon valves generate considerably higher pressures in infusion lines, especially at bolus flow rates, but such pressures alone are not high enough to trip pump occlusion alarms if set appropriately. Their presence would certainly reduce the threshold for other causes of raised line pressures being present before occlusion alarms are tripped. There are multi-infusion devices without anti-siphon valves that produce lower line pressures, but clinicians should be aware of the risk of siphoning when using them.
